# On the Epistemological Crisis in Genomics

**DOI:** 10.2174/138920208784139546

**Published:** 2008-04

**Authors:** Edward R Dougherty

**Affiliations:** Department of Electrical and Computer Engineering, Texas A&M University, Computational Biology Division, Translational Genomics Research Institute, USA

## Abstract

There is an epistemological crisis in genomics. At issue is what constitutes scientific knowledge in genomic science, or systems biology in general. Does this crisis require a new perspective on knowledge heretofore absent from science or is it merely a matter of interpreting new scientific developments in an existing epistemological framework? This paper discusses the manner in which the experimental method, as developed and understood over recent centuries, leads naturally to a scientific epistemology grounded in an experimental-mathematical duality. It places genomics into this epistemological framework and examines the current situation in genomics. Meaning and the constitution of scientific knowledge are key concerns for genomics, and the nature of the epistemological crisis in genomics depends on how these are understood.

## INTRODUCTION

There is an epistemological crisis in genomics. The rules of the scientific game are not being followed. Given the historical empirical emphasis of biology and the large number of ingenious experiments that have moved the field, one might suspect that the major epistemological problems would lie with mathematics, but this is not the case. While there certainly needs to be more care paid to mathematical modeling, the major problem lies on the experimental side of the mathematical-experimental scientific duality. High-throughput technologies such as gene-expression microarrays have lead to the accumulation of massive amounts of data, orders of magnitude in excess to what has heretofore been conceivable. But the accumulation of data does not constitute science, nor does the *a postiori* rational analysis of data.

The ancients were well aware of the role of observation in natural science. Reason applied to observations, not reason alone, yielded pragmatic knowledge of Nature. This is emphasized by the second century Greek physician Galen in his treatise, *On the Natural Faculties*, when, in regard to the effects of a certain drug, he refutes the rationalism of Asclepiades when he writes, “This is so obvious that even those who make experience alone their starting point are aware of it… In this, then, they show good sense; whereas Asclepiades goes far astray in bidding us distrust our senses where obvious facts plainly overturn his hypotheses” [1]. For the ancients, the philosophy of Nature might have dealt with principles of unity, ideal forms, and final causes, but natural science was observation followed by rational analysis. This was especially so during the Roman period, as evidenced by their remarkable engineering achievements.

The change brought about by the “new science” of the Sixteenth and Seventeenth Centuries is based on the integration of two principles: (1) design of experiments under constrained circumstances to extract specifically desired information; and (2) mathematical formulation of knowledge. The two principles arise from the two sides of the scientific problem, the source of knowledge and the representation of knowledge in the knower. Perhaps the greater revolution in knowledge is the design of experiments. One need only think of Archimedes’ mathematical analyses of fluidics and mechanics to see that the ancients recognized the central role of mathematics, even if they did not understand that role in the modern sense. But the modern concept of experiment is a different matter altogether. The Greeks understood the role of observation, but not the idea of a controlled scientific experiment. Nor was this idea familiar to Ptolemy. It was Galileo who realized that Nature should not be observed *au natural*, but instead should be artificially constrained to focus on the phenomena of interest without the effects of confounding variables. For modern science, reason does not enter the picture following observations; rather, it first provides a protocol for the observations so their analysis will characterize relations of interest and not be confounded by a multitude of secondary variables. For modern science, reason steps outside of Nature and constrains the manner in which Nature presents herself for analysis. While such constraint causes inexactitude relative to the knowledge of all variables and their interactions, Nature’s complexity precludes such full knowledge anyway. For modern science, reason brings focus to the scientific enterprise. 

Everything begins with the notion of a designed experiment – that is, methodological as opposed to unplanned observation. Rather than being a passive observer of Nature, the scientist structures the manner in which Nature is to be observed. The monumental importance of this change is reflected by the inclusion of the following statement concerning the early modern scientists, in particular, Galileo and Torricelli, by Immanuel Kant in the preface of the second edition of the *Critique of Pure Reason:*

They learned that reason only perceives that which it produces after its own design; that it must not be content to follow, as it were, in the leading-strings of Nature, but must proceed in advance with principles of judgment according to unvarying laws and compel Nature to reply to its questions. For accidental observations, made according to no preconceived plan, cannot be united under a necessary law… Reason must approach Nature… [as] a judge who compels witnesses to reply to those questions which he himself thinks fit to propose. To this single idea must the revolution be ascribed, by which, after groping in the dark for so many centuries, natural science was at length conducted into the path of certain progress [2].

A good deal of the crisis in genomics turns on a return to “groping in the dark.”

In previous papers, we have considered how the model-experiment duality leads to a contemporary epistemology for computational biology [3], treated the validation of computational methods in genomics [4], and characterized inference validity for gene regulatory networks in the framework of distances between networks [5]. Here we focus on how the experimental method leads to a general scientific epistemology and how contemporary genomic research often fails to satisfy the basic requirements of that epistemology, thereby failing to produce valid scientific knowledge. 

## SCIENTIFIC KNOWLEDGE

Experiments drive the epistemology of science. The product of an experiment is a set of measurements. These form the data of sensibility, the empirical (as opposed to a rational) basis for knowledge. In themselves, measurements do not constitute scientific knowledge. They must be integrated into a conceptual system. Scientific knowledge is constituted *via *synthesis of the observed measurements. These are related to variables and relations among the variables. A complex of variables and their relations compose a mathematical model. A scientific theory consists of two parts: (1) a *mathematical model* composed of symbols (variables and relations between the variables), and (2) a set of *operational definitions* that relate the symbols to data.

The model must be mathematical because it relates measurements *via *numerical concepts, such as length, weight, rate of decay, intensity, etc., or judgments *via *logical constructs. A basic model may be formed by some set of relations, say a stochastic model of a gene regulatory network, but knowledge does not stop there. Stopping there may make the system useless. Given some defining relations for a regulatory network, mathematical deduction leads to the full flowering of the knowledge inherent in the relations – for instance, deriving the steady-state distribution of the network. Indeed, if one wishes to use the network to obtain therapeutic strategies, then a natural way to proceed is to derive intervention policies that favorably alter the steady state of the system by reducing the long-run probability of the system being in an undesirable state. In Kantian terminology, the mathematical model *constitutes* the object of our knowledge. The experiment and the mathematical model form two inseparable requirements for scientific knowledge. Either without the other cannot yield scientific knowledge. Kant famously stated, “A concept without a percept is empty; a percept without a concept is blind” [2].

A mathematical model alone does not constitute a scientific theory. The model must be predictive. Mathematics is intrinsic because science is grounded in measurements; however, a model’s formal structure must lead to experimental predictions in the sense that there are relations between model variables and observable phenomena such that experimental observations are in accord with the predicted values of corresponding variables. These predictive relations characterize model validity and are necessary for the existence of scientific knowledge. In *The Rise of Scientific Philosophy*, Hans Reichenbach argues that reason supplies the predictive element in science:

If the abstract relations are general truths, they hold not only for the observations made, but also for observations not yet made; they include not only an account of past experiences, but also predictions of future experiences. That is the addition which reason makes to knowledge. Observation informs us about the past and the present, reason foretells the future [6].

This statement leads to the necessity of a predictive framework for validation. System validation requires that the symbols be tied to observations by some semantic rules that relate not necessarily to the general principles of the mathematical model themselves but to conclusions drawn from the principles. The conceptual system must be related to the experimental methodology. Phillipp Frank summarizes the situation both historically and epistemologically:

Reichenbach had explicitly pointed out that what is needed is a bridge between the symbolic system of axioms and the protocols of the laboratory. But the nature of this bridge had been only vaguely described. Bridgman [7] was the first who said precisely that these *relations of coordination* consist in the description of physical operations. He called them, therefore, *operational definitions* [8].

This means that the model be such that it can be tied to physical operations. Moreover, it leaves open the manner and the extent to which the model must be related to experimental outcomes. The general epistemological perspective seems clear, but its application to particular settings is not specified.

Where is the model to come from and how does one characterize model validity relative to a measurement process? Albert Einstein states,

In order that thinking might not degenerate into ‘metaphysics,’ or into empty talk, it is only necessary that enough propositions of the conceptual system be firmly enough connected with sensory experiences and that the conceptual system, in view of its task of ordering and surveying sense experience, should show as much unity and parsimony as possible. Beyond that, however, the system is (as regards logic) a free play with symbols according to (logically) arbitrarily given rules of the game [9].

According to Einstein, the model (conceptual system) is a creation of the “imagination.” The manner of this creation is not part of the scientific theory. The classical manner is that the scientist combines an appreciation of the problem with reflections upon relevant phenomena and, based upon mathematical knowledge, creates a model. As Einstein states, this creation is free except that it must conform to the rules of the mathematical game. At issue is what is meant by “enough propositions” being “firmly enough connected with sensory experiences.” Operational definitions are required, but their exact formulation in a given circumstance is left open. Their specification constitutes an epistemological issue that must be addressed in mathematical (including logical) statements. Absent such a specification, a purported scientific theory is meaningless. Reichenbach states, “The reference to verifiability is a necessary constituent of the theory of meaning. A sentence the truth of which cannot be determined from possible observations is meaningless” [6]. Because a model consists of mathematical relations and system variables must be checked against quantitative experimental observations, there is no nonmathematical way to describe the requirements and protocols to assess model validity.

Suppose a geneticist recognizes phenotypic effects from blocking the promoter region of a gene to prevent transcription or from using RNAi to suppress signaling. The geneticist might then propose a mathematical model of the form 

$\left(g\to 0\right)\Rightarrow \left(\mathrm{p1}\to \mathrm{p2}\right),$

where *g* → 0 means that the protein product of gene *g* never reaches its target, *p*1→ *p*2 means phenotype *p*1 is transformed to phenotype *p*2, and ⇒ is probabilistically interpreted as prediction. The model is validated by an experiment designed to reflect conditions under which the model is hypothesized. If the geneticist were to make observations without specifying a precise mathematical model (including a probability distribution to characterize the probabilistic aspects of the model) and a protocol for predictive validation, then there would be no scientific knowledge. 

The fundamental requirement of a scientific validation procedure is that it must be predictive. A scientific theory is not complete without the specification of achievable measurements that can be compared to predictions derived from the conceptual theory. Moreover, it depends on the choice of validity criteria and the mathematical properties of those criteria as applied in different circumstances. The sensory measurements and the manner in which they are to be compared to the conceptual system must be formally specified. The validity of a theory is relative to this specification, but what is not at issue is the necessity of a set of relations tying the conceptual system to operational measurements. It makes no sense to argue about the validity of a scientific theory without specifying the validation protocol. A scientific theory is inter-subjective, but the epistemological criteria underlying a particular validation are open to debate. Once the validation requirements are specified, the mathematical model (conceptual system) is valid relative to the validation criteria and to the degree that the requirements are satisfied, that is, to the degree that predictions demanded by the validation protocol and resulting from the mathematical model agree with experimental observations.

## LIMITS TO UNDERSTANDING

The dependence of science on experiment and prediction necessitates that scientific knowledge be constituted within mathematical systems, not ordinary language, because the latter is not conducive to rigorous probabilistic statements quantifying predictability. Common sense notions that play no role in a predictive model are not part of science, however useful they might be in everyday life. For instance, consider causality, which has deep roots in epistemology. In his *Physics*, Aristotle states, “Knowledge is the object of our inquiry, and men do not think they know a thing till they have grasped the ‘why’ of it (which is to grasp its primary cause). So clearly we too must do this as regards both coming to be and passing away and every kind of physical change” [10]. Aristotle is making the epistemological claim that to have knowledge of a physical change we must know its cause. Although “cause” is an everyday term that seems to be meaningful, the history of philosophy is strewn with attempts to define different types of causes and to make clear the notion of causality. But does such a common sense term with a long history in the discussion of natural phenomena have any scientific content?

Relative to modern science, perhaps the most important analysis of causality is due to David Hume. He notes that a cause and its effect are contiguous and related *via *temporal priority, with the cause prior to the effect, but more than contiguity and temporal priority, causality relates to a “necessary connection” between the cause and the effect and we come to this conclusion “when one particular species of events has always, in all instances, been conjoined with another” [11]. But what is the ground of this belief in causality? Hume points out that the principle of causality is neither intuitively certain nor provable by logical means, and that our belief in the principle rests not on reason, but on habit and custom. In *A Treatise of Human Nature*, he writes,

[The] supposition that the future resembles the past is not founded on arguments of any kind, but is derived entirely from habit, by which we are determined to expect for the future the same train of objects to which we have been accustomed…. All our reasonings concerning causes and effects are derived from nothing but custom and belief is more properly an act of the sensitive than of the cogitative part of our nature [12].

If causality rests on habit and custom, then to the extent that scientific knowledge requires causality, the ground of scientific knowledge is brought into question. Based on Hume’s analysis, there is no logical reason to accept the principle of causality, so that one may choose to accept or reject it. For Hume, the concept of a necessary connection between phenomena is subjective. If logical necessity is considered to be a requirement for knowledge, then science does not produce knowledge. 

Kant agrees with Hume that the principle of causality is not a scientific principle; however, whereas for Hume, habit underlies belief in causality, for Kant, causality is a form imposed on the data by the nature of the human mind. The mind imposes forms on the data of sensation, and scientific knowledge is limited by these forms. The way things appear, such as being spatially coordinated and connected by causality, are due to subjective *a priori* conditions for human knowledge. One cannot know things apart from the manner in which they conform to these *a priori* mental forms. While Kant differs from Hume on the ground of causality, for science, the basic point remains. Kant writes, “[Hume] justly maintains that we cannot comprehend by reason the possibility of causality, that is, of the reference of the existence of one thing to the existence of another, which is necessitated by the former” [13].

Hume pushes his analysis beyond causality itself, to the very relationship between observation and scientific theory when he states, “From the mere repetition of any past impression, even to infinity, there never will arise any new original idea, such as that of a necessary connection; and the number of impressions has in this case no more effect than if we confined ourselves to one only” [12]. If science rests on necessary connections – for instance, the certainty that event *B* will follow event *A* – then the ground of science is destroyed because certain knowledge about Nature is impossible, no matter how many times we observe a relation. The concept of induction as logic is demolished. There is no argument based on reason that allows one to assert a certain relation based on experience. Hume’s analysis shows that inductive inference is not logically necessary. Habit may lead one to conclude that a relation will hold the next time the antecedent is observed, but there is no logical certainty. 

Hume’s reasoning does not imply the end of science, but only that science needs an epistemology suitable to an empirical perspective. Its content and validity can not be based on a system suitable to abstract logic or mathematics, where propositions can be asserted to be either true or false. Science must differentiate itself from a metaphysical concept of knowledge that looks for connections beyond the observable. Reichenbach puts the matter in the following way:

Speculative philosophy is characterized by a *transcendental* conception of knowledge, according to which knowledge transcends the observable things and depends upon the use of other sources than sense perception. Scientific philosophy has constructed a *functional* conception of knowledge, which regards knowledge as an instrument of prediction and for which sense observation is the only admissible criterion of nonempty truth [6].

Scientific truth is pragmatic truth and this truth is contained in the predictive capacity of a scientific theory. Scientific knowledge is about the future. This pragmatism towards the future is bluntly affirmed by Feynman when he writes, “Knowledge is of no real value if all you can tell me is what happened yesterday” [14]. Past observations may lead one to construct a theory, say through statistical estimation, but the theory must predict the future. As stated by Riechenbach, “A mere report of relations observed in the past cannot be called knowledge. If knowledge is to reveal objective relations of physical objects, it must include reliable predictions. A radical empiricism, therefore, denies the possibility of knowledge” [6].

Prediction is not certitude. Instead of causality, science involves conditional distributions that describe the probability of a *target* random variable *Y* given the values of a set of *predictor* random variables, *X*_1_, *X*_2_,…, *X_m_*. In particular, given the predictor random variables, the best prediction (relative to mean-square error) for the value of *Y* is its conditional expectation. Causality is replaced by conditioning. Statements concerning conditional prediction can be validated *via *experimentation. The meaning of a statement can be rigorously defined within the framework of probability theory and its relation to measurable phenomena can be mathematically characterized within the theory of statistics. If the predictor variables are temporally antecedent to the variable to be predicted, then we have forward prediction. The terms “cause” and “effect” never appear because they lack empirical foundation. Erwin Schroedinger explains, “It can never be decided experimentally whether causality in Nature is 'true' or 'untrue.' The relation of cause and effect, as Hume pointed out long ago, is not something that we find in Nature but is rather a characteristic of the way in which we regard Nature” [15]. One may make a philosophic choice to view Nature causally, but this viewpoint lies outside of science.

As an illustration, it has been shown that experimentally increasing the levels of the Wnt5a protein secreted by a melanoma cell line *via *genetic engineering methods directly alters the metastatic competence of that cell as measured by the standard *in vitro* assays for metastasis [16]. A scientific statement may take the form of predicting the likelihood of metastasis conditioned on the state of the *WNT5A* gene or the level of the Wnt5a protein. Notice the quantification: there must be a probability of metastasis under some specified set of conditions, and the validity of the statement rests with the accuracy of that probability. It is alright for that probability to be different under different conditions, for instance, depending on the age or sex of the patient, but under each different condition, the validity is determined by the accuracy of the probability statement under that condition.

Because Hume was still thinking in the rationalist tradition while attacking a rationalist conception of science, he could claim that “the number of impressions has in this case no more effect than if we confined ourselves to one only.” If certainty must be obtained for valid knowledge, then his argument is sound; however, his reasoning does not apply to a probabilistic formulation of scientific knowledge because knowledge is constituted in the probability distribution of the random variables. For statistical inference, the accuracy of the distribution inferred from the data improves with the number of observations (under suitable assumptions on the sampling procedure). Hence, scientific knowledge is contingent because new data may change the model, which in this case is the inferred distribution. The contingency of scientific knowledge has long been recognized and predates Hume’s assault on induction. In the *Mathematical Principles of Natural Philosophy*, Isaac Newton writes, “In experimental philosophy we are to look upon propositions inferred by general induction from phenomena as accurately or very nearly true, notwithstanding any contrary hypothesis that may be imagined, till such time as other phenomena occur, by which they may either be made more accurate, or liable to exceptions” [17]. As the founder of mathematical physics, Newton appreciated the role of mathematics in science, but he also recognized contingency – that is, no necessary connection between past observations and the future. It is not that he rejected causality. Indeed, he writes, “We are to admit no more causes of natural things than such as both true and sufficient to explain their appearances…. Therefore in the same natural effects we must, as far as possible, assign the same causes” [17]. Rather, it his recognition that induction cannot with certainty reveal relations.

Even if we were to accept causality in the form of necessary connections, only if all causal factors were known could we predict effects with certainty. In complex situations, such as the regulatory system of a cell, one cannot conceive of taking account of all contributing factors. Model complexity is limited due to several factors, including mathematical tractability, data requirements for inference, computation, and feasible experimental design. Thus, there will be latent variables external to the model affecting the variables in the model and making the model behave stochastically. For instance, consider a situation where a set *M* of “master” genes deterministically controls a set *S* of “slave” genes. If only a proper subset N⊂M is in the model, then each configuration of the latent genes in *M* - *N* produces a *context* for the model. Model behavior changes with context changes because the slaves’ values depend on all genes in *M*, so that the manner in which the genes in *N* control the slaves relative to the model network depends on the latent genes in *M* - *N* [18]. For a fixed setting of the latent genes the masters in *M* exhibit deterministic control, but since the contexts change with the latent genes and these genes are not part of the network, the control internal to the model network is stochastic rather than deterministic. Whereas our previous discussion of contingency relates to the changing form of the model as new data are acquired, contextual changes introduce a second form of contingency, one in which at any given time the model is contingent upon the latent variables. If one is not careful – for instance, not observing a system sufficiently long or under sufficiently varied conditions – one might miss the latent effects and obtain a model that only works in restricted settings. This is fine, so long as those conditions are known, but they might well not be known on account of lack of information regarding the latent variables. A basic goal of experimental design is to minimize latent effects on the observations.

The truth of a scientific theory rests with its validation and a theory is validated independently of the thinking leading to it. No amount of rationalist explanation can validate a theory. Science is not about rationalist explanation, neither in its classic philosophic form of explaining events in terms of natural categories or its more recent computational form in terms of explaining the data by fitting a model. It is not unusual to hear it said that some theory “explains” some phenomena. One listens to the explanation and it all seems quite reasonable. The explanation fits the data. Consider the following statement of Steven Jay Gould: “Science tries to document the factual character of the natural world, and to develop theories that coordinate and explain these facts” [19]. Perhaps this statement would have been accurate during medieval times, but not today. While it is true that theories coordinate measurements (facts), it is not the documented measurements that are crucial, but rather the yet to be obtained measurements. Gould’s statement is *prima fascia* off the mark because it does not mention prediction. 

Science is not about data fitting. Consider designing a linear classifier. A classifier (binary decision function) is constructed according to some design procedure that takes into account its mathematical structure, the data, and its success at categorizing the data relative to some criterion. The result might be good relative to the assembled data; indeed, the constructed line might even classify the data perfectly. But this linear-classifier model does not constitute a scientific theory unless there is an error rate associated with the line, predicting the error rate on future observations. Of critical importance to the scientific epistemology is that the model, consisting of both classifier and error rate, is valid only to the extent that the reported error rate is accurate. A model is validated neither by the rational thinking behind the design procedure nor its excellent data-fitting performance. Only knowledge of its predictive power provides validity. In practice, the error rate of a classifier is estimated *via *some error-estimation procedure, so that the validity of the model depends upon this procedure. Specifically, the degree to which one knows the classifier error, which quantifies the predictive capacity of the classifier, depends upon the mathematical properties of the estimation procedure. Absent an understanding of those properties, the results are meaningless.

Confusion of the scientific method with explanation is perhaps the greatest impediment to appreciating the nature of science – for instance, in a statement like, “Science explains natural phenomena.” Under the word “explain” in *Webster’s Unabridged Dictionary*, one finds three modern usages: (1) to make plain, clear, or intelligible; to clear of obscurity, (2) to give the meaning or interpretation of; to expound, (3) to account for; to state reasons for [20]. All of these are applicable to ancient science but none of them describe modern science. Intelligibility entails the formulation of a conceptual system. If we assume that this means the formulation of a mathematical model when intelligibility is used in the context of science, then one can accept intelligibility as part of science. Meaning and interpretation are not relevant to science; rather, they are philosophical categories. Lastly, “accounting for” and “stating reasons for” also refer to philosophical discourse, although one might argue that a model fitted to data “accounts for” the data. In any event, the main point is that nowhere among these definitions of explanation is there a mention of a designed experiment or predictive validation. This is because an everyday word like “explain” carries with it an everyday meaning and science is not an everyday enterprise. 

Let us focus on Intelligibility, which may be the interpretation of explanation that is most often confused with science. If we take intelligibility to mean that the phenomena themselves are grasped by the intellect, then this would imply that Nature is accessible to the human intellect. It is true that the mathematical model (conceptual system) is intelligible, but that is because the mathematical model is constructed by humans in accordance with human intelligibility. But the model does not mirror the physical world. One might argue that what is meant by explanation is mathematical explanation, in the sense that the equations fit the observations. Even if we accept this data-fitting meaning of explanation, it leaves out the fundamental aspect of scientific meaning – prediction. 

The limits of ordinary understanding have become clearer during the Twentieth Century and, accordingly so, ordinary understanding cannot be a requirement for scientific knowledge. This point is strongly emphasized by Richard Feynman in the following statement made before beginning a series of lectures on quantum electrodynamics to an audience of non-specialists:

What I am going to tell you about is what we teach our physics students in the third or fourth year of graduate school — and you think I'm going to explain it to you so you can understand it? No, you're not going to be able to understand it…You see, my physics students don't understand it either. That is because I don't understand it. Nobody does... It is whether or not the theory gives predictions that agree with experiment. It is not a question of whether a theory is philosophically delightful, or easy to understand, or perfectly reasonable from the point of view of common sense. The theory of quantum electrodynamics describes Nature as absurd from the point of view of common sense. And it agrees fully with experiment. So I hope you can accept Nature as she is — absurd [21].

The absurdity is not intrinsic to Nature. Absurdity is a human category and the absurdity of Nature is relative to ordinary human understanding. The philosophical notion that the human mind has the capacity to understand Nature in everyday categories has gone by the wayside. Modern science is about prediction, not understanding. 

It is not that we are without any understanding whatsoever; as previously noted, we understand the mathematical model. Our knowledge of phenomena resides in the mathematical model, insofar as that knowledge is conceptual. But here we must avoid the danger of slipping into rationalism, mistaking the conceptual system for Nature herself. Scientific knowledge does not stop with reasoning about possibilities and creating a model. It goes further to include a predictive validation methodology and then actual validation. Reichenbach notes that “the very mistake which made rationalism incompatible with science” is “the mistake of identifying [scientific] knowledge with mathematical knowledge” [22]. It is here that we see a great danger lying in Gould’s formulation. Without operational definitions and concomitant experimental protocols for validation, as well as the validation itself, the development of “theories that coordinate and explain” facts quickly drifts into rationalism. Reasoning, either in the form of conceptual categories such as causality or *via *a mathematical system, is applied to data absent any probabilistic quantification relating to the outcome of future observation. Explanation and opinion replace scientific methodology. Whose reasoning do we trust? A formal validation procedure settles the matter.

Explanations might help one arrive at a mathematical model or give one satisfaction, but they are not part of scientific theory. This view is unacceptable to some. A striking current example is the intelligent design movement. William Dembski, a major proponent of that movement, writes, “Admitting design into science can only enrich the scientific enterprise. All the tried and true tools of science will remain intact. But design adds a new tool to the scientist’s explanatory tool chest” [23]. The problem here is that science has no “explanatory tool chest.” The scientist has a method. Dembski provides no mathematical model, no operational definitions, and no experimental protocol. In fact, he recognizes that intelligent design is not part of science, so he wants to return science to the domain of reasoning and explanation, where non-predictive arguments concerning complexity and design can be entertained. In the case of intelligent design, the return would be dramatic. The intelligent design argument is nothing but a re-surfacing of the classical physico-theological argument that was rejected as scientific by Kant in the late Eighteenth Century. 

## IS GENOMICS UNDERSTANDABLE

When he refers to Nature as being absurd, Feynman is not criticizing his understanding of the mathematical systems that allow one to model physical phenomena and to make predictions regarding those phenomena; rather, he is referring to a lack of categorical understanding of the physical phenomena themselves. Light is conceived as neither wave nor particle. Thus, the categorical requirement that it be one or the other is violated. From the Kantian perspective, the object of sensibility cannot be conformed to the categories of understanding and therefore cannot be understood. As a product of the human intellect, a mathematical model is *ipso facto* understandable. Nature is not a product of the human intellect. 

Although biology does not present us with the anomalies of quantum physics, the problem of understanding remains. The need for a systems-based approach, in particular, network modeling, has long been recognized in biology. In their famous 1946 paper, Norbert Wiener and Arturo Rosenblueth considered the properties of random nets of conducting fibers, which are used to help characterize fibrillation [24]. Regarding genomics, in reference to his seminal 1961 paper with Francois Jacob [25], Jacques Monod writes, “The logic of biological regulatory systems abides not by Hegelian laws but, like the workings of a computer, by the propositional calculus of George Boole [26]. In 1969, the use of logical relationships to characterize gene regulation was formalized in the Boolean-network model by Stuart Kauffman [27]. In the concluding remarks of his 1966 book, *Principles of Development and Differentiation*, Conrad Waddington points directly towards a mathematically rigorous systems theory when he writes,

In my opinion, at least, the three problems immediately in front of us are these: What is the nature of the change that renders a cell competent, so that it is ready to be switched into a particular developmental path? What is it that triggers off the switch and puts the cell into a state of determination, which is only with difficulty reversible, and can normally be transmitted through several cell generations? Finally, how are the activities of all the genes concerned in any developmental pathway tied together, so that they proceed in an integrated and orderly manner – or does this, perhaps, follow from the answers to the first two questions? [28].

The insights of Monod, Kauffman, and Waddington into the role of switching networks in biological regulation lead at once to the requirement that biological investigation depend on the theory of multivariate dynamical processes, which will of necessity be random processes on account of latent variables and inherent biological variability, and that there is no nonmathematical way to constitute biological knowledge. This conclusion is evident in Wiener’s description of his collaboration with Rosenblueth. In the original 1948 edition of *Cybernetics: or Control and Communication in the Animal and Machine*, Wiener states, “Thus, as far back as four years ago, the group of scientists about Dr. Rosenblueth and myself had already become aware of the essential unity of the set of problems centering about communication, control, and statistical mechanics, whether in the machine or in living tissue” [29].

Biological systems behave as multivariate random processes of interacting variables and this is the framework in which its laws must be formulated. In particular, gene regulatory modeling involves stochastic nonlinear dynamical systems. These may be continuous or discrete, and they can be synchronous or asynchronous. As in all modeling situations, the more detailed the model, the greater the computational complexity and the more difficult the inference from data. Given a network model, at least two basic issues arise: (1) the phenotypic issue – characterizing the steady-state behavior of the system, and (2) the translational issue – determination of control strategies to favorably alter the steady-state behavior of the system. It may be very difficult to characterize the steady-state distribution of the system in terms of system parameters. Even if this is done, can one really claim to have an understanding of the steady-state distribution in terms of sensory intuitions regarding the genes? Even under the coarsest quantization, a binary network, and only 10 genes, the transition probability matrix of a Markov regulatory model is 1024 × 1024 and this determines a steady-state distribution with 1024 states. One is often mystified at how small perturbations in the parameters dramatically alter the steady-state behavior. Typically, mathematical analysis in terms of low-order statistical characteristics of a dynamical process allows application of the system, but even then intuition of properties entailed by the covariance matrix is rare except in the case of very simple covariance structures. The dependency on mathematics and the lack of intuition are even more extreme when wants to use the regulatory model to determine optimal therapeutic policies [30]. Fundamental, and often difficult, mathematical analyses must be performed to arrive at control strategies, and these are especially involved if one wishes to achieve robust strategies not overly sensitive to system identification or imperfect application of control. There is no hope of obtaining categorical understanding of a policy’s performance by considering the phenomena themselves.

If human beings had sensory experience of traveling near the speed of light, then perhaps our ordinary understanding would grasp changing masses and clocks slowing or speeding up. If we had sensory experience at the quantum level, then perhaps we would display no surprise at the behavior of a photon in the famous double-slit experiment. Our difficulties of understanding arise because the categories of our ordinary understanding relate to possible sensory experiences. These difficulties extend to genomics. We have no sensory experience with networks of thousands of nonlinearly interacting nodes exhibiting feedback, distributed regulation, and massive redundancy. The reasons for lacking understanding are different from those in physics, but they are compelling in their own way. Nature is absurd from the human perspective because we lack the categories of understanding with which to intuit it – be it physics or biology. 

## THE CURRENT SITUATION IN GENOMICS

Almost from the onset of the high-throughput microarray era, papers reporting classifiers based on gene-expression features have appeared. There have also been cautionary warnings about the dangers of misapplication of classification methods designed for use with at most hundreds of features and many thousands of sample points to data sets with thousands or tens of thousands of features (genes) and less than one hundred sample points (microarrays) [31-32]. Keeping in mind the thousands of gene expressions on a microarray, consider a sampling of sample sizes for cancer classification: acute leukemia, 38 [33]; leukemia, 37 [34]; breast cancer, 38 [35]; breast cancer, 22 [36]; follicular lymphoma, 24 [37]; glioma, 50 (but only 21 classic tumors used for class prediction) [38]; and uveal melanoma, 20 [39]. This is a tiny sampling of the host of microarray classification papers based on very small samples and selecting feature sets from among thousands of genes. 

Since the foundation of scientific knowledge is prediction, the scientific worth of a classifier depends on the accuracy of the error estimate. If a classifier is trained from sample data and its error estimated, then classifier validity relates to the accuracy of the error estimate, since this estimate quantifies the predictive capability of the classifier. An inability to evaluate predictive power would constitute an epistemological barrier to being able to claim that a classifier model is scientifically sound. Certainly, there are mathematical issues at each step in applying classification to microarray data. Can one design a good classifier given the small samples commonplace in genomics? [40] Can one expect a feature-selection algorithm to find good features under these limitations? [41] These concerns, while important for obtaining useful classifiers, are epistemologically overridden by the concern that the predictive capability, and therefore the scientific meaning, of a designed classifier lies with the accuracy of the error estimate. Except in trivial cases, there has been no evidence provided that acceptable error estimation is possible with so many features and such small samples. Even worse, in many cases studied it has been shown to be impossible [42-45]. Hence, not only have the vast majority of the papers not been shown to possess scientific content, large numbers of them have been shown not to possess scientific content. Braga-Neto writes, “Here, we are facing the careless, unsound application of classification methods to small-sample microarray data, which has generated a large number of publications and an equally large amount of unsubstantiated scientific hypotheses” [40]. The failure of the research community to demand solid mathematical demonstrations of the validity of the classification methods used with the type of data available has resulted in a large number of papers lacking scientific content.

Many epistemological issues in genomics relate to statistics. Mehta *et al*. write, “Many papers aimed at the high-dimensional biology community describe the development or application of statistical techniques. The validity of many of these is questionable, and a shared understanding about the epistemological foundations of the statistical methods themselves seems to be lacking” [46]. They are calling attention to a lack of sound statistical epistemology, which renders the results meaningless. The point is further emphasized by Dupuy and Simon, who write, “Both the validity and the reproducibility of microarray-based clinical research have been challenged” [47]. To examine the issue, they have reviewed 90 studies, 76% of which were published in journals having impact factor larger than 6. Based on a detailed analysis of the 42 studies published in 2004, they report:

Twenty-one (50%) of them contained at least one of the following three basic flaws: (1) in outcome-related gene finding, an unstated, unclear, or inadequate control for multiple testing; (2) in class discovery, a spurious claim of correlation between clusters and clinical outcome, made after clustering samples using a selection of outcome-related differentially expressed genes; or (3) in supervised prediction, a biased estimation of the prediction accuracy through an incorrect cross-validation procedure [47].

The situation is actually much worse than stated here, since in high-dimensional, small-sample settings, cross-validation error estimation, which is ubiquitous in microarray studies, does not provide acceptable error estimation (as will be illustrated in the following paragraph) [42-45]. Thus, using cross-validation in supervised prediction undermines scientific validity.

The consequences of ignoring epistemology can be illustrated by considering gene-expression classification. As commonly practiced, a feature set is found from among thousands of genes *via *some feature-selection algorithm, a classifier based on this set is trained on a small sample of less than 100 microarrays, and its error is estimated using the training data, often by cross-validation, even though cross-validation possesses large variance when used with small samples [42]. The validity of the classifier model, which consists of classifier and error estimate, depends on the accuracy of the error estimate. Unfortunately, in such a scenario the estimated and true errors are often virtually uncorrelated, as we will now demonstrate with an example [45]. The data are from a micorarray experiment relating to lung cancer [48]. There are 203 tumors (microarrays), 139 adenocarcinoma and 64 other tumor types. The 2000 genes with the highest variance are used. On each trial, 50 microarrays are randomly chosen, a feature-selection algorithm (in this experiment, t-test feature selection) is used to select 20 genes and a 3-nearest-neighbor classifier is trained. The true error of the trained classifier is estimated using the 153 microarrays not selected. Given this large test set, the true error should be well-estimated and this estimate is taken as the true error. The training-data error estimate, which is what would be found in a 50-microarray experiment, is obtained using 5-repeat, 5-fold cross-validation. 10,000 independent trials are performed. The scatter plot of the cross-validation versus true-error pairs is shown in Fig. (**1**), together with the regression line. The line is almost horizontal, indicating virtually no regression of the true error on the estimated error. The correlation between the true and estimated errors is 0.04. This, together with the large spread of the scatter plot, shows that the estimated error is essentially useless in predicting the true error. Thus, the trained classifier model is meaningless!

Recognizing the risks of small-sample classifier design, authors have sometimes proposed using additional computational analyses to support a given classification result [49-50]. Unfortunately, the supporting methods themselves may not have been demonstrated to be informative. For instance, some papers suggest the use of permutation-based p values for obtaining information regarding the selection of relevant genes or for assessing the quality of classification. Essentially, a statistic relating to class discrimination is computed from the data, the class labels are randomized some large number of times, the statistic is computed for each re-labeling, a histogram is formed from these re-labeled statistics, and the *p* value of the statistic corresponding to the actual labeling is computed. The issue is whether this *p* value is informative. If the *p* value gives insight into the distribution of the error or the reliability of the estimated error, then an argument can be made for using the *p* value to assess classifiers. Since the randomly re-labeled data contain little or no information on the true joint distribution of the labels and the gene-expression levels, any insight based on the *p* value must come solely from the estimated error. To be specific, if ε_0_ and ε_1_ are the error estimates for the randomized and actual data, respectively, then *p* is the probability that ε_0_ ≤ ε_1_. Intuitively, the null hypothesis *H*_0_ is that the classifier does not discriminate and the alternative hypothesis *H*_1_ is that it does discriminate. The top part of Fig. (**2**) gives the *p* value as a function of the estimated error for the actual data and the bottom part gives the distribution of the error estimates, these being for 3-nearest-neighbor classification, sample size 40, leave-one-out cross-validation error estimation, and a Gaussian model for which the optimal classifier has error 0.10 [51]. Comparing the two parts of the figure, we see that, for the region where the mass of the error estimates lie, there is no regression of the *p* value on the error estimate. Thus, the *p* value says essentially nothing about the error and is therefore useless as a classifier performance measure.

Experimental design is a key element in drawing statistical conclusions. A properly designed experiment can substantially increase the power of the conclusions, whereas a poorly designed experiment can make it impossible to draw meaningful conclusions. Potter has drawn attention to this issue in the context of high-throughput biological data by distinguishing between mere observation and experimental design, the fundamental distinction between pre-modern and modern science:

Making the observations with new and powerful technology seems to induce amnesia as to the original nature of the study design. It is though astronomers were to ignore every thing they knew both about how to classify stars and about sampling methods, and instead were to point spectroscopes haphazardly at stars and note how different and interesting the pattern of spectral absorption lines were. Nonetheless, I doubt the astronomers would claim to be doing an experiment. This dilettante’s approach to either astronomy or biology has not been in vogue for at least half a century [32].

In fact, it has not been in vogue since Galileo and Torricelli. Are we to return to “groping in the dark?”

In this vein, the ubiquity of data mining techniques is particularly worrisome. These tend to search for patterns in existing data without regard to experimental design or predictive capability. Keller points out the danger of trying to draw grand inferences from patterns found in data. Referring to William Feller’s classic text [52] on probability theory, she writes,

By 1971, the attempt to fit empirical phenomena to such distributions was already so widespread that Feller felt obliged to warn his readers against their overuse....Feller’s emphasis on the logistic curve as ‘an explicit example of how misleading a mere goodness of fit can be’ was motivated precisely by the persistence of such ‘naïve reasoning’ [53].

Data mining is often erroneously identified with pattern recognition when, in fact, they are very different subjects. Pattern recognition can be used as a basis for science because it is based on a rigorous probabilistic framework [54]. On the other hand, all too often, data mining techniques consist of a collection of computational techniques backed by heuristics and lacking any mathematical theory of error, and therefore lacking the potential to constitute scientific knowledge.

While inattention to epistemology in genomic classification is troubling, the situation with clustering is truly astounding. As generally practiced, there is no predictive aspect and hence no scientific content whatsoever. Indeed, Jain *et al*. state that "clustering is a subjective process,” [55] so that it lacks the basic scientific requirement of inter-subjectivity. In the context of genomics, Kerr and Churchill have asked the epistemological question: “How does one make statistical inferences based on clustering” [56]. Inferences are possible when clustering is put on a sound probabilistic (predictive) footing by recognizing that, whereas the epistemology of classification lies in the domain of random variables, [54] the epistemology of clustering must lie within the framework of random sets [57]. A great deal of study needs to be done in this direction before clustering can practically provide scientific knowledge. In the mean time, so-called “validation indices” are sometimes used to support a clustering result, but these are often poorly correlated to the clustering error and therefore do not provide scientific validation [58].

Epistemological considerations for genomics inexorably point to systems biology. It would seem obvious that systems biology should be based on systems theory, which, as we have discussed, is a direction clearly pointed to a half century ago in the work of Wiener, Rosenblueth, Monod, Waddington, Kauffman, and others. It is the approach taken in genomic signal processing, where both the dynamics of gene regulatory networks and their external control are being pursued within the context of systems theory [59]. Genomic research has mostly taken a different path. Based upon the historical path of genomics, Wolkenhauer goes so far as to virtually cleave genomics from systems biology when he writes,

The role of systems theory in systems biology is to elucidate the functional organization of cells. This is a complementary but very different effort to genomics, biophysics, and molecular biology, whose primary role it has been to discover and characterize the components of the cell – to describe its structural organization. A basic philosophical point systems theory makes is that objects and relations between objects have the same ontological status. Life is a relation among molecules/cells and not a property of any molecule/cell; a cell is built up of molecules, as a house is with stones. A soup of molecules is no more a cell than a plane is a heap of metal [60].

Wolkenhauer is making an empirical observation regarding a widespread inattention to systems theory. Genomics, being the study of multivariate interactions among cellular components, requires systems-based modeling, in particular, the use of nonlinear stochastic dynamical systems, whether these be in the form of differential equations, discrete networks, Markov processes, or some other form of random process. Science and engineering have more than half a century of experience with stochastic systems. Since it is impossible to conceive of modern communication and control systems absent their being grounded in systems theory, it is surely impossible to conceive of meaningful progress in genomics without the use (and extension of) this theory. Of course, there are obstacles. Experiments need to be designed and carried out in a manner suitable for the construction of nonlinear dynamical systems and systems theory needs to be developed in ways appropriate to biological modeling [61]. These are imposing tasks. Nonetheless, based on our long experience with humanly designed systems it is virtually certain that the study of biological systems cannot meaningfully progress without well thought out experiments and deep mathematics. 

## CONCLUSION

Is the epistemological crisis in genomics critical or topical? I believe it is topical. New models and perhaps new mathematics will be required, but there is no need to alter the Twentieth Century scientific epistemology. If one disagrees, then he or she must propose a different epistemology and seek to justify it prior to making any scientific claims. For instance, those who argue that it is scientifically legitimate to apply error estimation rules whose properties are unknown are in the position of having to deny the fundamental role of prediction in science. Unless one is willing to return to medieval thinking, denial of prediction as the operational ground of science would require the formulation of a new ground upon which to relate events. Denial of the role of mathematics as the carrier of scientific knowledge would require the introduction of another kind of language in which to make scientific statements precise, inter-subjective, and quantifiable. Denial of the role of designed experiments aimed at extracting specific information would mean a return to the “groping in the dark” of pre-Galilean science. And denial of the requirement for operational definitions would sunder measurements from reason and lead to a form of neo-rationalism that, in Einstein’s words, would degenerate into “empty talk.”

## Figures and Tables

**Fig. (1):**
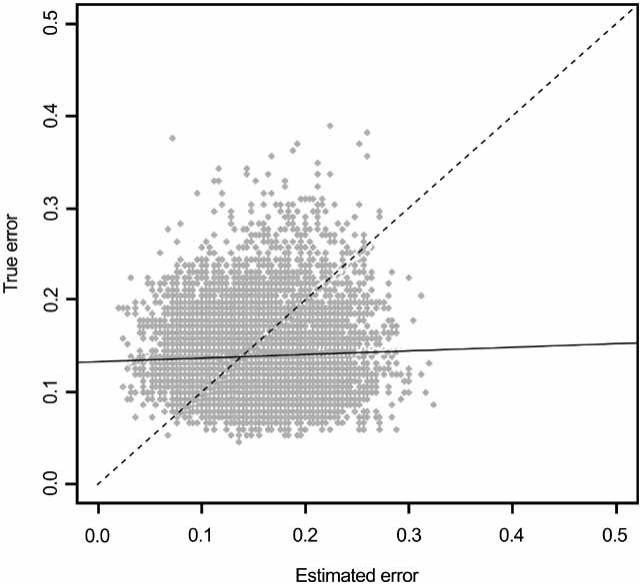
Regression of the true error on the estimated error.

**Fig. (2):**
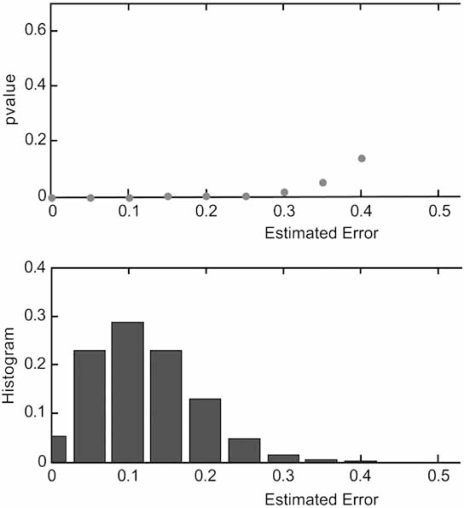
Regression of the permutation *p* value on the estimated error: Top part: *p* value as a function of the estimated error for actual data. Bottom part: error distribution,
